# Natural resonance frequency of the brain depends on only intracranial pressure: clinical research

**DOI:** 10.1038/s41598-020-59376-7

**Published:** 2020-02-13

**Authors:** Tetsuya Goto, Kenji Furihata, Kazuhiro Hongo

**Affiliations:** 1 Department of Neurosurgery, Shinshu University School of Medicine, Matsumoto, Japan; 2 Department of Neurosurgery, St. Marianna University School of Medicine, Kawasaki, Japan

**Keywords:** Neurophysiology, Experimental models of disease

## Abstract

To understand and control intracranial pressure (ICP) is required for treatments in various clinical situations. To establish non-invasive ICP prediction method, we focused on the natural resonance frequency (NRF) of the brain. The ICP value, pulse waveform of intracranial pressure (PWICP) and cervical carotid pulse waveform (CCPW) were simultaneously collected from patients who underwent neurosurgical treatment. A total of 43 data were obtained from 27 patients. The total measured time was 29,653 seconds and the measured mean ICP value in each data ranged from 3.82 to 69.39 (mean 25.9) hPa. Respiratory synchronized cardiac pulses were selected and following CCPW and PWICP were collected. The transfer characteristics from collected CCPW to PWICP were calculated. The initial negative peak was judged as the NRF of the brain. The relationship between the ICP value and the NRF of the brain was presented on the quadratic functions graph (ICP = 0.0329(NRF)^2^ + 0.0842NRF; R^2^ = 0.9952). It means that the individual NRF only depends on their ICP value. The ICP value will be predicted by checking NRF of the brain from somewhere.

## Introduction

The need to understand and control intracranial pressure (ICP) is required for treatments in clinical neurosurgery, neurology and emergency medicine. The ICP value can only be measured by direct intracranial catheter placement, for example, through ventricular drainage or an ICP sensor^1–3^. Transcranial Doppler ultrasonography^4^, tympanic membrane displacement^5,6^, and optic nerve sheath diameter^7^ have been investigated for non-invasive ICP monitoring, A preliminary report of the HS-1000 (HeadSense Medical Ltd.), in which the HS-1000 evaluated an acoustic signal from the ipsilateral ear including a short beep from the contralateral ear, has possibility of non-invasive ICP monitoring^8^. On the other hand, Raboel *et al*. stated that they were inaccurate and could not be used as an alternative to invasive techniques^9–11^. Recent well-designed research also presented that the ICP could not be predicted by tympanic membrane pressure^6^.

Our motivation for this research is to predict the ICP value by analysing the extra-auditory canal pressure waveform (EACPW). It is considered that the EACPW includes the component of ICP value because of their anatomical connection^5,6,12–16^. We theorised that the ICP value could be predicted with the use of the transfer function method from the cervical cardiac pulse waveform (CCPW) to EACPW^12^. To predict the ICP value from EACPW, for which there is no information of the direct current components included, we focused on the natural resonance frequency (NRF) of the brain. The particular structure has its own NRF, which depends on its consistency. The brain is in an individual NRF and the NRF correlates with the ICP; resonance phenomenon of the brain can be observed with the bump of the arterial pulse in every cardiac pulse. Ommaya *et al*. observed low-range natural frequencies (5–10 Hz) in the rotational brain motion of primates and humans in 1960 era^17^. System transfer function analysis of intracranial cavity has been studied in animal model since 1980 era^18–21^. By extent information about NRF of the brain, idea of non-invasive ICP prediction which was based on human brain tissue resonance frequency correlation with ICP value has been proposed by Michaeli D. *et al*.^22^. In recent papers^23,24^, although it is considered that the NRF of the brain is determined by the brain weight and CSF volume compliance, the theory was based on the MRI findings. The pulse waveform of intracranial pressure (PWICP) and CCPW have not been analyzed in individual clinical patients.

We discovered a simple relationship between the ICP value and the NRF of the brain by the transfer function method using the CCPW and PWICP from our clinical data. By utilising this algorithm, the individual ICP value can be predicted by a patient’s NRF. Our results and the mechanism of the relationship between the ICP value and the NRF of the brain are discussed in this report.

## Materials and Methods

### Patient criteria

The ICP value, PWICP and CCPW were simultaneously collected from patients who had undergone ventricular drainage or ICP sensor placement for medical treatment at Shinshu University Hospital from November 2014 to March 2017. All experiments were performed in accordance with relevant guidelines and regulations. The measurement of this information was approved by the Ethics Committee of Shinshu University School of Medicine “Development of non-invasive intracranial pressure monitoring” approved number 2343. Informed consent was obtained from all the patients or their families. Some participants under the age of 18 years, informed consent has been obtained from a parent.

A total of 27 patients, 17 men and 10 women, were included in the present research. The age of the patients ranged from 14–83 (mean 54) years, body height of 137–187 (mean 163) cm and body weight 32–103 (mean 61) kg. Of the 27 patients six had a presentation of trauma, four had glioma, five had an extra-axial tumour, four had intracerebral haemorrhage, three had infarction, four had hydrocephalus and one had a cerebrospinal fluid leakage. The ICP was measured by an ICP sensor in four patients and ventricular drainage in 23 patients (Table 1).

**Table 1:** ICP: intracranial pressure, MCA: middle cerebral artery, ASDH: acute subdural hematoma, ICH: intracerebral hematoma.

Materials							
Patient	Age (year)	Sex	Body height (m)	Body weight (kg)	Diagnosis	ICP measurement	Data number
1	42	w	1.65	41	glioma	drainage	1
2	51	w	1.58	58	glioma	drainage	2_1
							2_2
3	52	m	1.58	62	MCA occlusion	drainage	3_1
							3_2
4	49	w	1.52	59	meningioma	drainage	4_1
							4_2
5	67	m	1.65	60	hydrocephalus	drainage	5
6	29	m	1.7	57	hydrocephalus	drainage	6_1
							6_2
							6_3
7	23	m	1.65	63	ASDH	ICP sensor epidura	7_1
							7_2
							7_3
							7_4
8	47	m	1.75	66	ICH	ICP sensor brain	8_1
							8_2
							8_3
9	55	m	1.7	103	brain contusion	drainage	9
10	53	m	1.69	77	vestibular schwannoma	drainage	10
11	42	m	1.87	74	hydrocephalus	drainage	11
12	62	w	1.56	49	ICH	drainage	12
13	14	m	1.71	55	glioma	drainage	13
14	75	w	1.55	50	ASDH	ICP sensor epidura	14
15	62	w	1.37	32	hydrocephalus	drainage	15
16	54	m	1.72	71	brain contusion	ICP sensor epidura	16_1
							16_2
							16_3
							16_4
17	77	w	1.42	61	ASDH	drainage	17_1
							17_2
18	20	w	1.63	67	vestibular schwannoma	drainage	18
19	64	w	1.54	40	meningitis	drainage	19
20	69	m	1.81	82	MCA occlusion	drainage	20_1
							20_2
							20_3
21	77	m	1.6	60	MCA occlusion	drainage	21
22	83	w	1.4	39	subarachnoid hemorrhage	drainage	22
23	68	m	1.71	59	meningioma	drainage	23
24	67	m	1.64	53	ICH	drainage	24
25	17	m	1.73	61	glioma	drainage	25
26	74	m	1.68	77	brain contusion	drainage	26
27	74	m	1.67	63	CSF leakage	drainage	27

### Recording the data

The ICP value and PWICP were measured by either ventricular drainage or an ICP sensor. The ventricular drainage, in which 0 cmH_2_O height was adjusted at the level of the extra-auditory canal, was connected to the pressure sensor (SCK-7604 transducer blood sampling set: Argon Medical Devices: Frisco TX, USA). The ICP sensor (Integra Camino^®^ Intracranial Pressure Monitor: Integra LifeSciences Corporation: Plainsboro NJ, USA) was placed at the subdural space or intra-parenchymal portion. CCPW is pulse tonometry. Original sensor was made by approximately 2*10^−6^ m^3^ volume of polyethylene airtight cylinder. The cylinder, which base was flat and round 8 mm in diameter, was placed on the neck at the position of the cervical carotid artery and fitted by a soft band. A pressure change in the cylinder was measured by high sensitive microphone (EM215 Electric Condenser Microphone: Primo Corporation: Tokyo, Japan).

**Figure 1:**
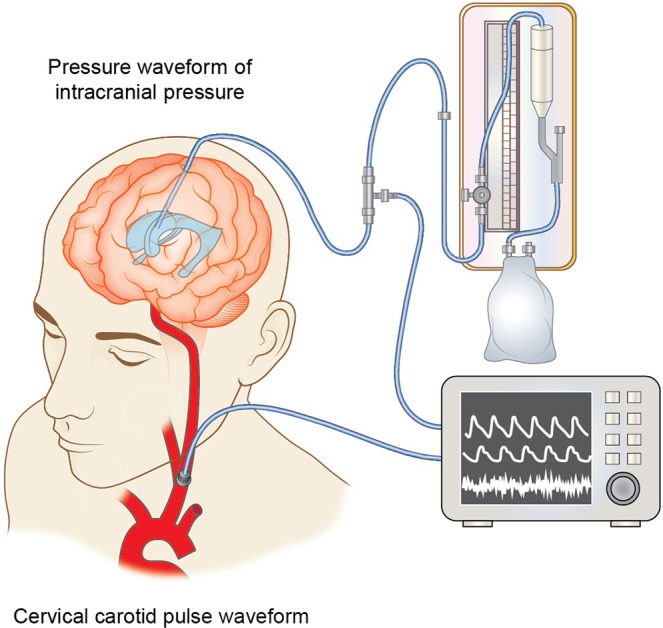
A conceptual schema of data sampling. Cervical carotid pulse waveform and pressure waveform of intracranial pressure were collected and stored simultaneously in 200 Hz by original device.

All the information was collected and stored simultaneously in 200 Hz by original device made by Ichikawa Electric Corp. Ltd. Tokyo, Japan (Fig. 1).

In some patients measurements were undertaken on different days as different data.

### Analysing the data

**Figure 2:**
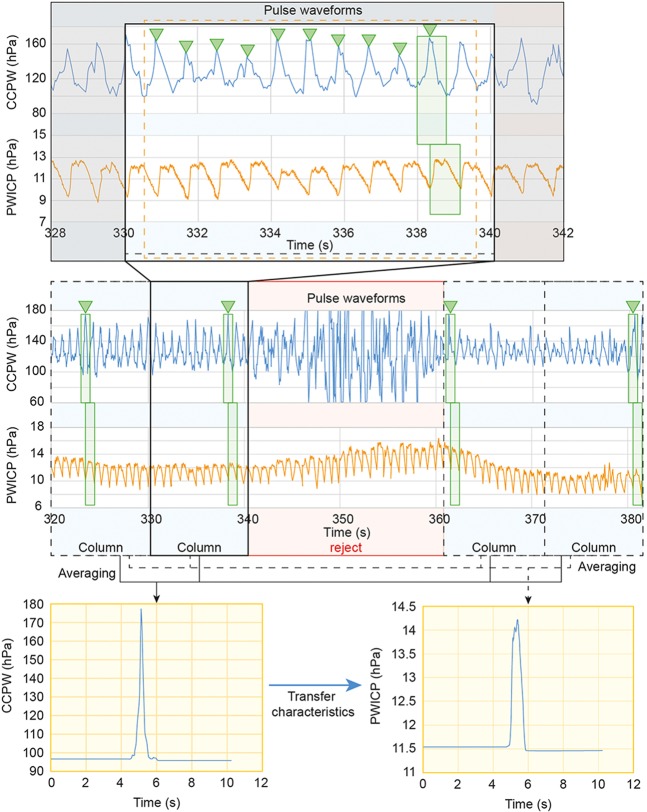
How to calculate the transfer characteristics between cervical carotid pressure waveform and pulse waveform of intracranial pressure. Center graph: The raw data of cervical carotid pulse waveform (CCPW) and the pulse waveform of intracranial pressure (PWICP). After rejection of the noise (red line square), the data was cut in consecutive 10.24 seconds which was called a “column” (black dot square). Upper graph: magnified one column. The largest one (green square) from the positive peak of cardiac pulsation of CCPW (green arrowhead) was selected in each column, as well as an synchronized PWICP. Lower two waveforms: The selected CCPW and PWICP were averaged by the weighted average method. Left panel: averaged CCPW after pressure transmission to the CCPW by the substitution of systolic/diastolic blood pressure value from the upper arm, Right panel: averaged PWICP. And the transfer characteristics from CCPW to PWICP was calculated.

**Figure 3:**
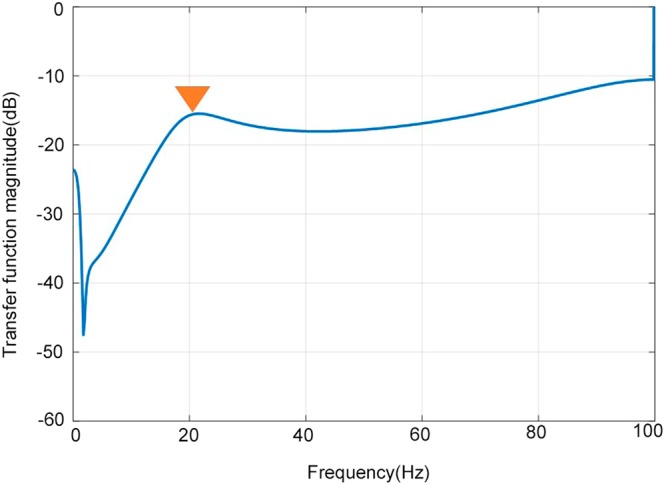
The graph for the relationship between transfer function magnitude and frequency. The yellow arrow indicates the NRF of the brain.

The data was analysed in an off-line state. All noise as a result of patient motion, artifact, or inappropriate sensor setting were rejected before analysis. (1) After rejection of the noise, the consecutive data was cut into 10.24 seconds, which was called a “column”. Each column covered a 5.12-second width. (2) The largest positive peak of cardiac pulsation of CCPW was picked-up from each column, as well as one synchronized PWICP. (3) The picked-up CCPW and PWICP were averaged using the weighted average method. (4) The transfer characteristics from CCPW to PWICP were calculated using the transfer function method, after pressure transmission to the CCPW by the substitution of systolic/diastolic blood pressure value from the upper arm (Fig. 2). (5) The NRF of the brain was calculated value as initial pole of the transfer characteristics. Figure 3 graph represented the relationship between transfer function magnitude and frequency. The NRF of the brain (yellow arrow) located near the first negative peak in the graph. (6) The ICP value was averaged in the whole of the selected columns, and the relationship between the ICP value and the NRF of the brain was compared.

### Calculation of transfer characteristics between CCPW and PWICP

The relationship was verified by Matlab version 2018b (The Mathworks, Natick, MA, USA). Transfer function models described the relationship between the inputs and outputs of a system using a ratio of polynomials. The model order was equal to the order of the denominator polynomial. The roots of the denominator polynomial were referred to as the model poles. The roots of the numerator polynomial were referred to as the model zeros. The parameters of a transfer function model were its poles, zeros and transport delays.

Where, *PWICP*(*s*), *CCPW*(*s*) and *noise* (*error: E*(*s*)) represented the Laplace transforms of the output, input and noise, respectively. In continuous-time, a transfer function model had the form:

$$PWICP(s)=\,\frac{num(s)}{den(s)}{e}^{-s\tau }CCPW(s)+E(s).$$

Numerator: *num*(*s*) and denominator: *den*(*s*) polynomials that defined the relationship between the input and the output. $\tau$ represented the delay. The choice of a model order was also affected by the amount of delay. The first step, a good idea of the input delay simplified the key task of matching up the rise points between the averaged CCPW and PWICP (See Figs. 2–3).

If we took the Z-transform, where, *PWICP*(*z*^*−1*^), *CCPW*(*z*^*−1*^) and *E*(*z*^*−1*^) represented the Z-transforms of the output, input and noise, respectively. ${z}^{-1}$ was the Z-transform of the lag operator. The transfer function had the form:

$$PWICP\,({z}^{-1})=\frac{num\,({z}^{-1})}{den\,({z}^{-1})}CCPW\,({z}^{-1})+E\,({z}^{-1}),$$

$$num\,({z}^{-1})={b}_{0}+{b}_{1}{z}^{-1}+{b}_{2}{z}^{-2}+{b}_{3}{z}^{-3}+\cdots +{b}_{n}{z}^{-n},$$

$$den\,({z}^{-1})=1+{a}_{1}{z}^{-1}+{a}_{2}{z}^{-2}+{a}_{3}{z}^{-3}+\cdots +{a}_{n}{z}^{-n}.$$

where, np was number of poles and nz was number of zeros in the estimated transfer function. Manually matching complicated features in the Bode plot (magnitude and phase data) of the frequency response of the high-order brain system was difficult to match with conventional first and second order numerators and denominators. The next task was to identify the orders. This method often required manual tuning of weighting parameters. Therefore, we checked the first resonance frequency, the Q (quality factor) and the fit for all 243 combinations of the np changed from three to eight, the nz changed from one to seven and the fine adjustment delays changed from zero to eight.

The fit showed that normalized root mean squared error (NRMSE) expressed as a percentage, defined as:

$$FitPercent=100\,(1-\frac{||PWIC{P}_{measured}-PWIC{P}_{\mathrm{mod}el}||}{||PWIC{P}_{measured}-\overline{PWIC{P}_{measured}||}}).$$

Where, $PWIC{P}_{measured}$ was the measured PWICP data, $\overline{PWIC{P}_{measured}}$ was its mean, $PWIC{P}_{model}$ was the simulated response of the model, and $||\cdots ||$ indicated the 2-norm of a vector. *FitPercent* varies between –Inf (bad fit) to 100 (perfect fit). If the value is equal to zero, then the model is no better at fitting the measured data than a straight line equal to the mean of the data.

From the calculated results, three functions of model order could be determined by analysing the improvement in the first resonance frequency (nearest neighbour search of the theoretical NRF), the Q (0.5 < Q < 10)^25^ and the fit (fit > 60%).

### Statistical analysis

We tried to model the obtained data using a second-degree polynomial function:

$$ICP={c}_{2}NR{F}^{2}+{c}_{1}NRF+{c}_{0}.$$

The unknown coefficient, *c*_0_, *c*_1_ and *c*_2_ were computed by minimizing the sum of the squares of the deviations of the data from the model (least squares fit). For the first step we used “polyfit” to find the polynomial coefficients. Matlab calculated the polynomial coefficients in descending powers. In the second step we used “polyval” to evaluate at the measured NRF. On the third step we identified the statistical determination between the variables *ICP* and *NRF* to justify modelling the data. A data model explicitly described a relationship between predictor and response variables. Linear regression fitted a data model that was linear in the model coefficients. The most common type of linear regression was a least squares fit, which would fit both polynomials. One method to find the better fit was to calculate the coefficient of determination, *R*^2^. *R*^2^ was one measure of how well a model would predict the data and fell between zero and one. The higher the value of *R*^2^, the better the model was at predicting data.

Where $\widehat{ICP}$ represented the calculated values of *ICP* and $\overline{ICP}$ was the mean of *ICP*. *R*^2^ was defined as:

$${R}^{2}=1-\frac{{\sum }_{i=1}^{n}{(IC{P}_{i}-{\widehat{ICP}}_{i})}^{2}}{{\sum }_{i=1}^{n}{(IC{P}_{i}-\overline{ICP})}^{2}}.$$

### Ethics

The simultaneous measurement of intracranial pressure and cervical carotid artery pressure waveform was approved by Ethical Committee of Shinshu University School of Medicine. “Development of non-invasive intracranial pressure monitoring” approved number 2343. https://www.shinshu-u.ac.jp/faculty/medicine/common/docs/i-rinri/r-shounin25.pdf (in Japanese).

## Results

The data were measured at one time in 18 patients, two times in eight patients, three times in three patients and four times in two patients. A total of 43 data were available from 27 patients and the total measured time was 29,653 seconds (measured time in each data: min. 256, max. 2,562, mean: 690 sec.). The total valid data time (measured time - reject time) was 19,256 seconds (valid time in each data: min. 102, max. 872, mean: 470 sec.). The total number of columns was 3,800 (number of columns in each data: min. 20, max. 170, mean: 93). The measured mean ICP value in each data was from 3.82 to 69.39 (mean: 25.9) hPa (Tables 2 and 3).

**Table 2:** ICP: intracranial pressure.

Data number	Measured data						
	Measured time	Reject time	Valid data time	Number of columns	ICP value	ICP value STD	Blood pressure (sistoric/diastoric)
	(s)	(s)	(s)	(Column=10.24 s)	(hPa)	(hPa)	(hPa)
1	272	47	225	44	31.08	4.22	160/93
2_1	319	19	300	60	9.66	1.57	170/93
2_2	701	199	502	98	19.40	3.51	146/93
3_1	1631	1529	102	20	35.56	12.01	239/81
3_2	295	85	210	41	16.50	8.63	186/106
4_1	697	259	438	85	13.60	1.08	181/100
4_2	736	335	401	78	13.72	2.62	173/93
5	867	398	469	91	19.27	1.12	190/101
6_1	2562	1905	657	128	36.69	4.50	173/88
6_2	1183	349	834	163	40.17	8.52	180/100
6_3	653	131	522	104	9.31	1.89	170/93
7_1	413	20	393	76	44.23	0.63	198/100
7_2	368	0	368	71	45.17	0.40	184/106
7_3	444	20	424	82	30.98	0.18	166/93
7_4	661	68	593	115	44.08	3.80	180/113
8_1	359	38	321	62	36.49	1.36	201/78
8_2	333	10	323	63	37.41	1.63	216/81
8_3	643	57	586	114	62.54	4.80	216/106
9	300	0	300	58	6.24	0.30	190/82
10	438	139	299	58	13.42	0.92	186/126
11	671	168	503	98	13.28	3.49	173/93
12	1292	803	489	134	10.57	3.64	160/80
13	856	432	424	82	21.57	2.52	146/67
14	949	77	872	170	69.39	1.22	186/93
15	957	348	609	119	15.89	2.01	146/80
16_1	1222	710	512	100	44.71	0.94	173/93
16_2	549	20	529	103	33.21	0.80	186/100
16_3	472	336	136	26	46.31	0.55	173/88
16_4	256	10	246	48	13.38	0.19	160/100
17_1	733	0	733	143	21.66	1.02	193/82
17_2	418	0	418	81	16.37	0.14	180/106
18	329	174	155	42	11.25	5.08	146/93
19	923	282	641	125	15.25	2.19	146/59
20_1	711	236	475	92	28.86	0.66	146/67
20_2	377	20	357	69	32.51	0.98	160/93
20_3	460	27	433	84	22.26	0.57	166/93
21	689	10	679	132	10.76	2.98	166/105
22	570	360	210	41	15.19	1.33	186/106
23	545	205	340	66	13.28	1.73	160/93
24	834	180	654	127	30.46	2.64	213/128
25	402	12	390	76	38.13	1.38	160/80
26	875	172	703	137	22.82	2.16	186/106
27	688	207	481	94	3.82	1.27	173/93

**Table 3:** NRF: natural resonance frequency.

Data number	Caliculated results					
	Number of poles	Number of zeros	Delay	Q	%fit	Calcurated NRF
	(np)	(zp)			(%)	(Hz)
1	6	4	8	0.97	92.95	30.20
2_1	8	6	4	2.91	90.34	15.85
2_2	6	1	3	6.33	83.38	23.55
3_1	6	5	6	1.67	64.63	31.53
3_2	7	1	0	4.94	61.15	20.29
4_1	6	1	4	5.44	64.64	19.47
4_2	8	3	7	0.75	73.99	19.74
5	8	1	1	3.03	67.63	23.02
6_1	8	7	2	6.50	85.92	33.15
6_2	7	2	5	4.28	82.44	33.55
6_3	5	1	3	3.75	83.95	16.56
7_1	7	3	2	5.24	62.37	36.48
7_2	6	2	4	1.92	64.75	36.44
7_3	7	5	4	2.71	61.35	29.87
7_4	7	2	7	5.17	64.51	35.10
8_1	6	3	6	5.08	62.56	32.25
8_2	7	3	0	0.61	75.59	31.40
8_3	5	1	5	3.32	63.8	42.51
9	5	3	7	2.36	61.57	13.80
10	4	3	3	2.00	61.77	18.27
11	7	6	2	1.51	88.78	19.22
12	7	3	3	3.08	62.46	15.42
13	7	5	6	2.05	68.17	24.75
14	7	6	2	3.71	87.47	45.38
15	7	1	1	4.07	76.54	20.97
16_1	6	2	6	3.20	81.54	35.61
16_2	6	3	8	3.67	82.11	30.88
16_3	7	4	8	4.37	83.11	36.56
16_4	6	5	3	1.62	84.47	19.79
17_1	5	3	0	1.51	63.83	23.08
17_2	7	4	8	2.89	62	22.11
18	7	3	2	2.03	76.69	17.27
19	5	4	6	0.84	88.31	20.17
20_1	8	3	7	3.74	84.94	28.94
20_2	6	2	3	1.46	91.63	30.35
20_3	7	2	8	3.23	89.09	24.80
21	7	3	0	1.38	77.37	17.90
22	6	5	6	3.09	71.66	20.80
23	6	5	2	1.65	79.97	19.00
24	5	1	1	5.87	67.45	29.59
25	6	2	1	1.01	63.08	33.09
26	3	2	2	2.57	76.3	26.47
27	8	1	0	1.25	76.6	10.71

**Figure 4:**
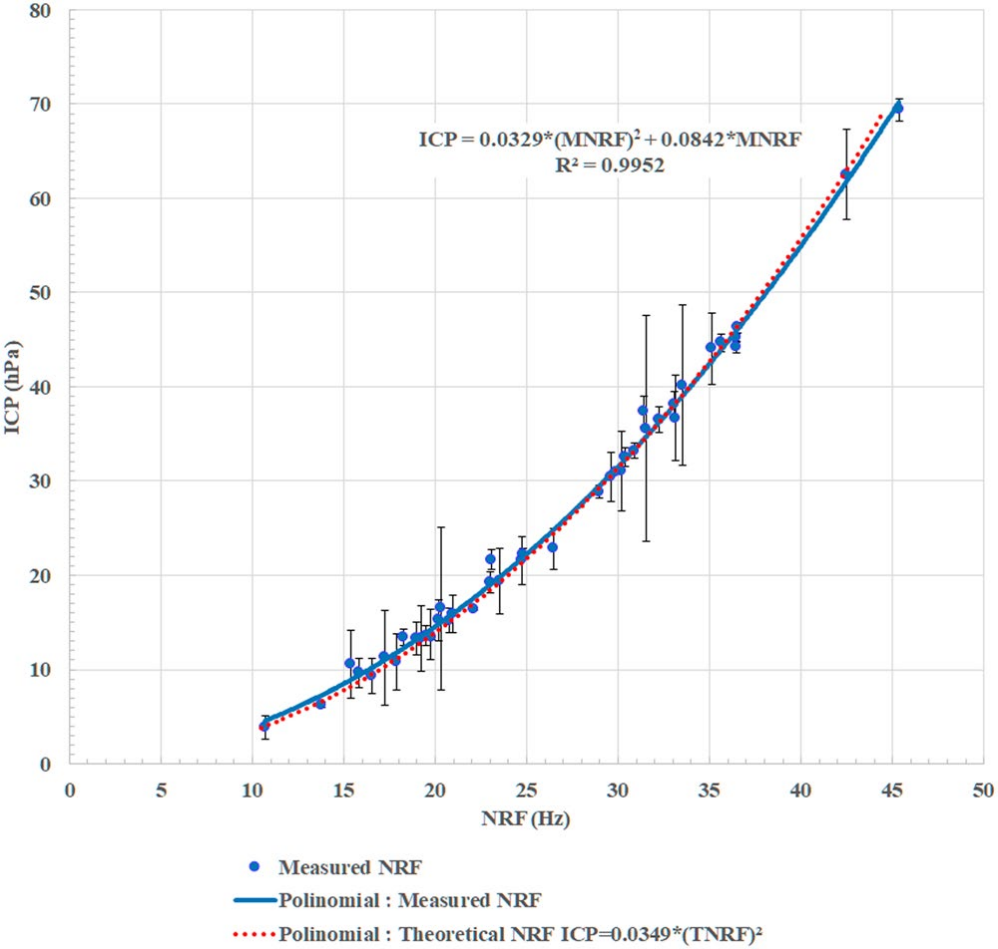
The graph for the relationship between the ICP value and the NRF of the brain. Blue points indicate data number (n = 40). Error bars indicate SD. These parameters are strongly correlated with each other (R2 = 0.9998). The blue polynomial: approximation graph from blue points. The red polynomial: theoretical calculation graph from discussion.

The ICP value and the NRF of the brain from 41 data were plotted in Fig. 4.

Every data was presented on the quadratic functions graph (ICP = 0.0329(NRF)^2^ + 0.0842NRF). These parameters strongly correlated with each other (R^2^ = 0.9952 coefficient of determination).

## Discussion

Many individual parameters may contribute to the NRF of the brain, such as intracranial volume, arterial wall stiffness, age, disease, blood pressure, blood concentration, cardiac output, general peripheral vessel resistance and body position. Furthermore, fluctuation of CCPW, ICP value and PWICP in every cardiac pulse makes it difficult to analyse. The respiratory rhythm is one of the most significant factors to modulate the ICP value, in which the modulation ratio of PWICP power spectral is over 55%^26^. The intrathoracic pressure decreases in exhalation and increases in inspiration. The jugular venous pressure is dependent on the intrathoracic pressure and the intracranial pressure is depending on the jugular venous pressure. We utilised the weight averaging method for respiratory synchronization in which the largest amplitude of CCPW in the 10.12 second column was picked-up. By the averaged CCPW and PWICP without respiratory modulation, the calculated NRF of the brain and measured ICP value were presented on the quadratic functions graph. This means that individual parameters without respiratory modulation are not related to the NRF determination.

### Theoretical analysis for NRF of the brain

**Figure 5:**
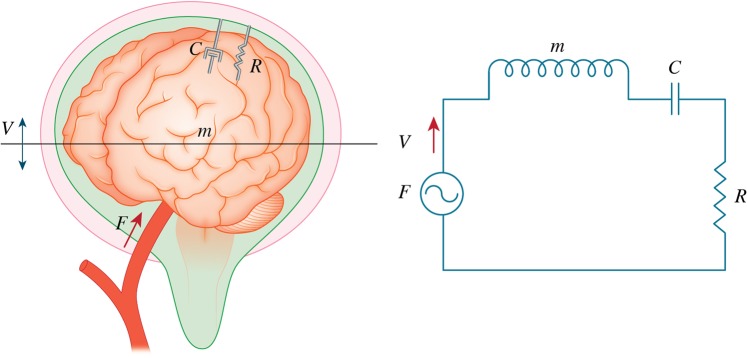
Theoretical analysis of the resonance phenomenon in the skull. Left: the schema of the simple brain acoustic dynamics model. The brain (weight: m kg) is located in the closed space. The CSF interferes with the movement of the brain by the effect of a shock absorber (cerebrospinal volumetric compliance: C F) and viscosity (resistance: R Ω). The arterial pulse (force: f N) moves the brain. The moving speed of the brain is velocity: v m/s. Right: mechanodynamically electrically equivalent circuit (weight: m kg) (condenser: C F) (resistance: R Ω) (force: f N).

This relationship between the ICP value and the NRF of the brain can be explained by the simple brain acoustic dynamics model (Fig. 5 left). The brain (weight: *m* kg) is located in the closed space and the CSF interferes with the movement of the brain by the effect of a shock absorber (cerebrospinal volumetric compliance: *C* F) and viscosity (resistance: *R* Ω). The arterial pulse (force: *F* N) moves the brain. The moving speed of the brain is velocity: *V* m/s. Each factor can be described as:

$${f}=Rv+m\frac{dv}{dt}+\frac{1}{C}\int vdt$$

These components can be changed in the electric circuit (Fig. 5 right).

If the arterial pulse can be substituted by a sigmoid curve, the NRF of the brain: *f*_0_ (Hz) can be described as:

$${{\rm{f}}}_{0}=\frac{1}{2\pi \sqrt{mC}}$$

By this theory, the NRF of the brain is only dependent on brain weight and cerebrospinal volumetric compliance. The average brain weight is 1.4 kg^27^. Cerebrospinal volumetric compliance is inversely proportional to the ICP value^25,28,29^. When the ICP is 13.33 hPa, the compliance was 0.0000473^30^; the coefficient: *C* of the cerebrospinal compliance can be calculated by:

$$C=\frac{0.0000473}{(\frac{ICP}{13.33})}$$

and the theoretical calculated relationship between ICP and the NRF is described as shown in Fig. 3 (red dots). Although the graph was introduced by substituting the previous proposed data into the simple brain mechanical model, our clinical data was strongly correlated (R^2^ = 0.9999 coefficient of determination and the standard error between the polynomial curves is 0.55 hPa). The strong correlation between the ICP value and the NRF of the brain from individual data means that the ICP value can be predicted from the NRF of the brain.

### Limitation

To understand the details of the relationship between the ICP value and the NRF of the brain, further data collection is necessary. Especially data for high ICP values, which is lacking in our research. Because increased ICP value remarkably decreases inflow of the blood to the intracranial space, the drastic change around the “non-filling” situation occurs. The adaptable range of cerebral autoregulation is different in each patient. Our result cannot adjust in very high range of ICP patient. We have no data on the small brain weights of paediatric and senile patients. External decompression surgery might affect our results. Radiological evaluation is also necessary.

### Future perspective

The correlation between the ICP value and the NRF of the brain may be utilised in the prediction of the ICP value. If the NRF of the brain can be recorded from somewhere, we can predict the ICP value. Theoretically the NRF can be transmitted and measured from various places, such as the movement of the tympanum, external ear pressure waveform and the thickness of the retinal membrane. Of course, these data must include not only the NRF of the brain, but also other resonance and oscillation frequency from other organs. If we can extract this specific frequency from extracranial organs, non-invasive intracranial pressure prediction will be possible.

## Conclusions

The individual NRF, which was calculated by the transfer function method using their CCPV and PWICP, only depends on the measured ICP value from ventricular drainage or an ICP sensor, based on the simple quadratic functions as presented. This means that individual parameters, without respiratory modulation, are not related to the NRF determination. This relationship could be explained by a simple brain mechanical model. The ICP value might be predicted by some non-invasive methods using this principle because the NRF consists of relative value.
